# The Beneficial Effects of Antifreeze Proteins in the Vitrification of Immature Mouse Oocytes

**DOI:** 10.1371/journal.pone.0037043

**Published:** 2012-05-23

**Authors:** Jun Woo Jo, Byung Chul Jee, Chang Suk Suh, Seok Hyun Kim

**Affiliations:** 1 Department of Obstetrics and Gynecology, Seoul National University Bundang Hospital, Seongnam, Korea; 2 Department of Obstetrics and Gynecology, Seoul National University College of Medicine, Seoul, Korea; 3 Department of Obstetrics and Gynecology, Seoul National University Hospital, Seoul, Korea; 4 Institute of Reproductive Medicine and Population, Medical Research Center, Seoul National University Seoul, Korea; Baylor College of Medicine, United States of America

## Abstract

Antifreeze proteins (AFPs) are a class of polypeptides that permit organismal survival in sub-freezing environments. The purpose of this study was to investigate the effect of AFP supplementation on immature mouse oocyte vitrification. Germinal vesicle-stage oocytes were vitrified using a two-step exposure to equilibrium and vitrification solution in the presence or absence of 500 ng/mL of AFP III. After warming, oocyte survival, *in vitro* maturation, fertilization, and embryonic development up to the blastocyst stage were assessed. Spindle and chromosome morphology, membrane integrity, and the expression levels of several genes were assessed in *in vitro* matured oocytes. The rate of blastocyst formation was significantly higher and the number of caspase-positive blastomeres was significantly lower in the AFP-treated group compared with the untreated group. The proportion of oocytes with intact spindles/chromosomes and stable membranes was also significantly higher in the AFP group. The AFP group showed increased Mad2, Hook-1, Zar1, Zp1, and Bcl2 expression and lower Eg5, Zp2, Caspase6, and Rbm3 expression compared with the untreated group. Supplementation of the vitrification medium with AFP has a protective effect on immature mouse oocytes, promoting their resistance to chilling injury. AFPs may preserve spindle forming ability and membrane integrity at GV stage. The fertilization and subsequent developmental competence of oocytes may be associated with the modulation of Zar1, Zp1/Zp2, Bcl2, Caspase6, and Rbm3.

## Introduction

Oocyte cryopreservation is an important strategy of fertility preservation. The improvement of oocyte vitrification protocol has significantly increased oocyte survival and contributed to increased pregnancy rates [1]–[5]. To date, the majority of successful assisted pregnancies have involved frozen and subsequently thawed mature oocytes that were collected after ovarian stimulation. However, ovarian stimulation with gonadotropins may not be suitable for many women who need to preserve their fertility, especially patients with breast cancer or other hormone-dependent cancers, or patients who require immediate chemotherapy. When there is not enough time for ovarian stimulation and/or when stimulation should be avoided, immature oocytes can be collected from the ovaries without hormonal stimulation. In most fertility centers, retrieved immature oocytes are usually allowed to mature *in vitro* and then vitrified at metaphase II [6], [7]. However, vitrification at the germinal vesicle (GV) stage is an alternative strategy that can prevent spindle depolymerization. These immature oocytes are arrested in the diplotene stage of prophase I. Theoretically, the use of immature GV-stage oocytes reduces the risk of polyploidy and aneuploidy because the chromosomes are diffuse and surrounded by a nuclear membrane [8], [9]. Although this method could enhance the GV survival rate, the maturation, fertilization, and subsequent embryonic development up to the blastocyst stage are impaired; this is considered the main problem associated with GV oocyte vitrification [10]–[12]. Therefore, more progress of oocyte developmental stage is needed to enhance the developmental potential of vitrified GV oocytes to ultimately achieve good clinical results.

Antifreeze proteins (AFPs) are present in the tissue and blood of Antarctic fish, insect, and plant. AFPs permit the survival of these animals and plants in sub-freezing environments [13]. Currently, three types of AFPs have been identified, each with a unique MW and amino acid sequence [13]. In addition, at least four different AFPs were described in teleost [14]. The benefits of AFPs on the cryopreservation of animal cells and organs have been demonstrated [13], [15]. However, Payne et al. reported adverse effects of AFP and AFGP supplementation after ram spermatozoa cryo-teatment [16]. Our preliminary study indicated that high dose of AFP supplementation (such as 0.5–1.0 mg/mL) also noted harmful effects on oocyte survival. The concentration (500 ng/mL AFP) was determined by premilinary experiments, where the concentration showed higher cleavage and blastocyst formation rates.

There were no studies that have investigated the effect of AFPs on the outcome of GV oocyte vitrification. AFPs are thought to kinetically depress the temperature at which ice crystals form and thus potentially prevent thermal shock. This mechanism may protect cell membranes against cold-induced injury [13], [17].

In the present study, we applied type III AFP during the vitrification of mouse GV-stage oocytes. We investigated whether AFP could protect against cryo-induced damage by evaluating the function and development of AFP-treated vitrified oocytes. In addition, we investigated whether gene expression related to oocyte function and development was affected by AFP supplementation.

## Results

**Figure 1:**
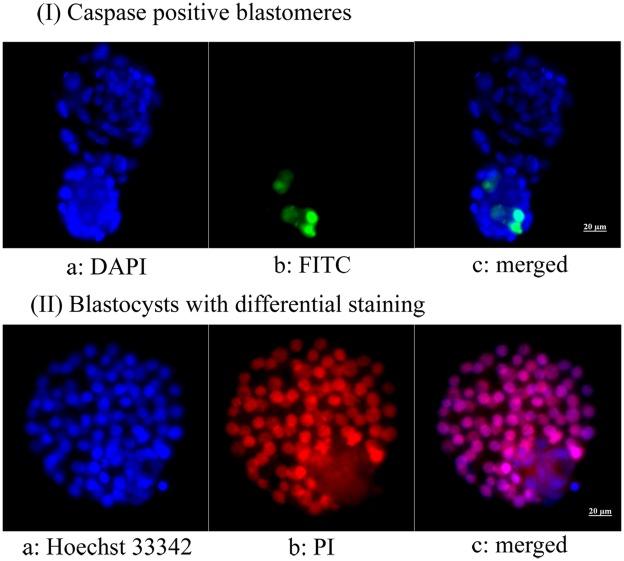
Microphotographs showing fluorescent caspase staining and differential staining of blastocysts derived from vitrified-warmed immature mouse oocytes. (I) Apoptotic blastomeres appear green; (II) red-pink indicates trophectoderm and blue indicates inner cell mass (400×). (I) a: DAPI, b: FITC, C: merged; (II) a: Hoechst 33342, b: propidium iodide, c: merged.

As shown in Table 1, the survival rate in the AFP-treated group was significantly higher than that in the untreated group. After IVM, the maturation rate in the AFP-treated group was similar to that in the untreated group. When IVF was performed, cleavage and blastocyst formation rates were significantly higher in the AFP-treated group. Total blastomere and TE and ICM cell counts were also significantly higher in the AFP-treated group. In addition, caspase positivity was significantly lower in the AFP-treated group (Figure 1).

**Table 1:** Development of vitrified-warmed germinal vesicle (GV)-stage oocytes with or without antifreeze protein (AFP; 500 ng/mL) supplementation in vitrification media (seven replicates).

	AFP-treated	Untreated	P
Initiated GV oocyte	171	178	
Survived	166 (97.1%)	162 (91.0%)	0.017
Matured	129 (77.7%)	126 (77.8%)	NS
Cleaved	118 (91.5%)	88 (69.8%)	<0.01
Blastocyst (per cleaved embryo)	96 (81.4%)	59 (67.1%)	0.019
Blastocyst (per initiated GV)	96 (56.1%)	59 (33.2%)	<0.01
Blastomere count per blastocyst	78.2±11.8a	67.8±11.3b	<0.01
No. of caspase-positive blastomeres	285/3,987 (7.1%)	363/3,188 (11.4%)	<0.01
No. of trophectoderms perblastocyst	69.6±11.8c	54.0±5.3d	<0.01
No. of inner cell masses perblastocyst	13.9±2.2c	11.4±1.9d	<0.01

Mean±SD.

Number of blastocysts tested: a = 51, b = 47, c = 23 d = 22.

**Figure 2:**
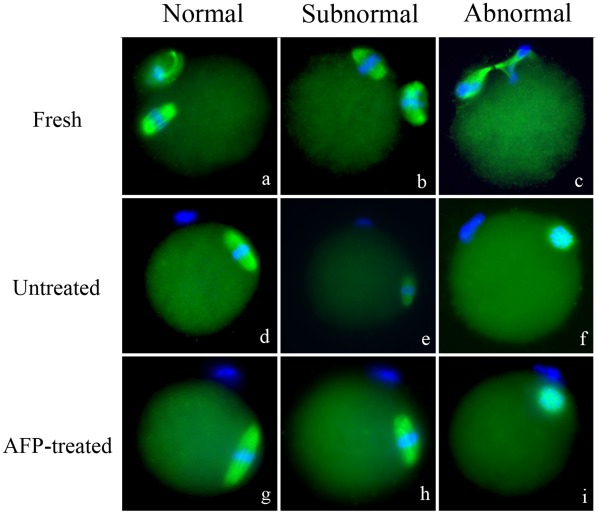
Representative microphotographs showing morphology of meiotic spindle organization and chromosome alignment in vitrified-warmed oocytes. AFP  =  Antifreeze protein. (x400).

**Figure 3:**
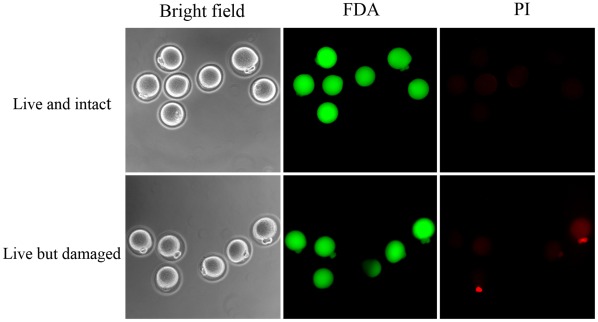
Vitrified-warmed oocytes stained with 1 µg/ml fluorescein diacetate (FDA, green) and 50 µg/ml propidium iodide (PI, red). Upper panel; live and intact oocytes showing positive FDA and negative PI. Under panel; live but damaged oocytes showing positive FDA and PI.

The grades of spindle and chromosome were divided into three groups; normal, sub-normal, and abnormal (Figure 2). A significantly higher proportion of oocytes with normal spindle and chromosome morphology was observed in the AFP-treated group (Table 2). In membrane integrity test, live and intact oocytes were observed more frequently in the AFP-treated group (92.1% vs. 75.0%; p<.05). The proportion of live oocytes with damaged membranes was significantly lower in the AFP-treated group (7.9% vs. 25.0%; p<.05) (Figure 3).

**Table 2:** Meiotic spindle organization and chromosome alignment of vitrified-warmed mouse oocyte supplemented with antifreeze protein (Five replicates).

	Untreated	AFP-treated	P
No. of oocytes examined	88	78	
Normal	53 (60.2%)	61 (78.2%)	0.013
Sub-normal	21 (23.9%)	8 (10.3%)	0.021
Normal+Subnormal	74 (84.1%)	69 (88.5%)	NS
Abnormal	14 (15.9%)	9 (11.5%)	NS

Mad2 expression was increased in the AFP-treated group compared with the fresh control or untreated groups. In contrast, Eg5 expression was markedly lower in the AFP-treated group. The levels of Hook1, Zar1, and Zp1 were higher in the AFP-treated group than in the untreated group. However, the levels of Hook1, Zar1, and Zp1 in the AFP-treated group were similar to those in the fresh control group. The expression of Zp2 was down-regulated in the AFP-treated group. Mater, Hsf1, Sod1, Zp3, and CIRP levels were similar in all three groups.

In AFP-treated oocytes, Caspase6 and Rbm3 were expressed at levels similar to those observed in fresh control oocytes, and lower than those observed in non-treated controls. Both vitrification groups showed relatively increased Caspase3 and decreased Bcl2 expression compared with the fresh control group. Bax expression was similar in all three groups.

## Discussion

Cryopreservation at the GV stage has merits. At this stage, chromosomes are inactive, and the complete barrel-shaped spindle has not formed yet. [8], [9]. Enhancing vitrification protocol at GV stage might be a potential method from the perspective of preserving spindle forming ability. We assumed that properly organized GV oocyte vitrification protocol helps to preserve normal spindle forming ability after warming.

Previously, we demonstrated that AFP could improve the developmental competence of vitrified-warmed mouse MII oocytes (Jo et al., 2011). In the present work, a significantly higher proportion of oocytes with intact spindle and chromosomal morphology was observed in the AFP-treated group compared with the untreated group. This result suggests that AFP supplementation could help preserve spindle/chromosome dynamics during immature oocyte vitrification and subsequent *in vitro* maturation. Our findings are consistent with other reports indicating that AFPs are able to protect oocyte membranes [13], [18]. This result could partly explain the improved survival, fertilization and subsequent embryonic development observed in the AFP-treated group.

It has previously been demonstrated that cryopreservation can modulate the expression of multiple genes within the oocyte [19]. We tested the expression of three genes involved in spindle integrity. As a spindle checkpoint protein, Mad2 regulates anaphase onset and genome integrity in the oocyte [20], [21]. Several previous reports suggested that Mad2 could be activated under stressful conditions, such as those induced by heat and cold [22], [23]. In the present study, the numbers of transcript of Mad2 was increased in AFP-treated oocytes compared with untreated controls. We speculate that the activation of Mad2 may be correlated with prompt spindle reassembly.

Eg5 is a plus-end-directed kinesin-related motor protein (KRP) that was previously shown to be involved in the assembly and maintenance of the mitotic spindle. *In vitro* experiments demonstrated that reduced or increased expression of Eg5 results in cell cycle arrest, defective centrosome separation, monopolar spindle formation [24]–[27], genomic instability and tumor formation in mice [28]. Our results indicated that Eg5 transcript abundance was elevated in the untreated group compared with the fresh control and AFP-treated groups, both of which showed similar Eg5 levels.

Hook1 plays a role in configuring the microtubule cytoskeleton and in regulating chromosome segregation. This protein is necessary for the correct positioning of microtubule structures within haploid germ cells [29], [30]. Thus, any alteration in Hook1 transcription may lead to chromosomal abnormalities [30]. In the present study, Hook1 expression was significantly down-regulated in the untreated group. However, AFP-treated and fresh control oocytes showed similar Hook1 mRNA levels. Collectively, increased Mad2 and Hook1 and decreased Eg5 expression might contribute to the preservative effect of AFP on spindle integrity during vitrification.

During folliculogenesis and oogenesis, oocytes accumulate maternal gene products, which are essential for supporting early embryonic development. Maternally derived mRNAs and proteins are produced and stored in the oocyte during oocyte growth and maturation. In mice, the roles of Mater, Hsf1, and Zar1 have been investigated [31]–[33]. The disruption of Mater in embryos results in arrest at the 2-cell stage [31]. In Hsf1 mutants, embryos are arrested at the 1-cell stage, and in Zar1 mutants, PN stage arrest occurs [32], [33]. In the present study, the levels of Mater and Hsf1 transcripts were similar across the three groups. Therefore, the expression of these two genes may not be affected by vitrification or AFP supplementation. However, Zar1 expression was down-regulated in the untreated group. This result suggests that increased fertilization competence might be correlated with Zar1 expression in the AFP-treated group.

Sod1 is an antioxidant enzyme that probably plays a crucial role in protecting embryos against oxygen toxicity *in vivo* and *in vitro* [34]. In our work, Sod1 expression was similar in all three groups. Therefore, this gene appears not to be affected by vitrification or AFP supplementation.

The zona pellucida (ZP) is a thick extracellular coat that surrounds all mammalian eggs. It consists of three glycoproteins, Zp1, Zp2 and Zp3 & [35], [36]. Zp3 serves as a primary sperm receptor and acrosome reaction-inducer; Zp2 serves as a secondary sperm receptor during fertilization [37]; and Zp1 is important for zona pellucida assembly around growing oocytes [35]. In the absence of Zp1 expression, females exhibited reduced fertility. In the present study, Zp3 expression was similar in all three groups. Zp2 was expressed at relatively lower levels in the AFP-treated group compared with both the fresh and untreated groups. In addition, Zp1 was down-regulated in the untreated group, which may be associated with lower fertilization rates in the untreated group.

In the current study, we evaluated the expression of four apoptosis-related genes, Bcl2, Bax, Caspase3 and Caspase6. Bcl2 is anti-apoptotic and promotes cell survival [38]. In contrast, Bax is pro-apoptotic and accelerates cell death [39]. Effector caspases, such as Caspase3 and 6, are expressed during the final step of apoptosis [40], [41]. In our study, Bcl2 expression was down-regulated in both vitrification groups. However, the AFP-treated group showed a relatively intense Bcl2 signal compared with the untreated group. Bax was expressed at similar levels in the three groups and thus does not appear to be affected by vitrification or AFP supplementation. Caspase3 was up-regulated in both vitrification groups, and AFP supplementation did not affect its expression. However, Caspase6 expression was increased in the untreated group compared with the fresh and AFP-treated groups. Taken together, these results suggest that vitrification *per se* may affect Bcl2 and Caspase3 expression, and AFP supplementation may decrease Caspase6 expression.

The expression of the cold injury-related genes CIRP and Rbm3 was examined to investigate the cryoprotective nature of AFP. CIRP is a recently identified cold-inducible RNA-binding protein that is induced at 32°C in mouse somatic cells *in vitro* [42], [43]. In the present study, CIRP expression was similar in the three groups. The change in temperature during *in vitro* culturing may have contributed to this result. However, Rbm3 was up-regulated in the untreated group and down-regulated in the AFP-treated group. The decrease in expression of Rbm3 may be one mechanism by which AFP protects oocytes from chilling injury.

In conclusion, we have demonstrated for the first time that AFP exerts positive effects on mouse GV-stage oocyte vitrification. The addition of AFP into vitrification media improved the cryo-survival, fertilization and subsequent embryonic development of immature mouse oocytes. AFP may exert its cryo-protective effect via mechanisms that stabilize spindle morphology and membrane integrity. The preservation of the spindle apparatus after IVM appears to be modulated by Mad2, Eg5 and Hook1. In addition, the enhanced fertilization and developmental competence of oocytes may be enhanced through a modulation of the expression of Zar1, Zp1/Zp2, Bcl2, Caspase6 and Rbm3.

## Materials and Methods

### Animals

Four- to five-week-old female BDF-1 mice (Orient Co., Seoul, Korea) were used for these experiments. Animal care was carried out in accordance with the guidelines established by the Institutional Animal Care and Use Committee (IACUC) of Seoul National University of Bundang Hospital. IACUC specifically approved this study. Approval number was 63-2010-021.

### Retrieval of Immature Oocytes

Mice were treated with i.p. injections of 5 IU PMSG (Sigma-Aldrich, St. Louis, USA). Mice were killed by cervical dislocation 48 hrs later, and both ovaries were excised and placed in 1 mL of washing medium (modified mouse tubal fluid, mMTF) supplemented with 0.4% (w/v) bovine serum albumin (BSA, Sigma). Cumulus-oocyte complexes (COCs) covered with compact cumulus cells were collected by puncturing the antral follicles.

### Vitrification and Warming of COCs

Oocytes were vitrified with equilibrium solution (ES) and vitrification solution (VS) in the presence or absence of 500 ng/mL AFP type III (A/F Protein Inc., Waltham, USA) using a CryoTop device. The concentration of AFP was determined by pilot experiments, which showed higher survival and blastocyst formation rates at 500 ng/mL AFP. The oocytes were suspended in an ES containing 7.5%(v/v) EG, 7.5%(v/v) PROH, and 20%(v/v) FBS (Invitrogen) in HEPES-buffered TCM-199 medium for 5 minutes. The oocytes were then transferred to VS containing 15% (v/v) EG, 15% (v/v) PROH, 0.5 mol/L sucrose, and 20% (v/v) FBS in TCM-199 for 45–60 seconds at room temperature. Five to six oocytes were loaded onto a CryoTop (Kitazato) which was then immediately plunged into liquid nitrogen for storage.

For warming, the CryoTop was immersed directly in a 37_C warming solution (containing 1.0 mol/L sucrose in 20% FBS–supplemented TCM-199) for 1 minute. The warmed oocytes were transferred to 0.5 mol/L and 0.25 mol/L sucrose in 20% FBS–supplemented TCM-199 for 3 minutes, respectively, and then washed twice with washing medium (20% FBS in TCM-199) before they were transferred to culture medium at 37_C in 5% CO2 in humidified air. Survival rate of the oocytes was assessed 1 hour after incubation. Survived oocytes were identified by the morphologic appearance of membrane integrity and discoloration of the ooplasm. The surviving oocytes were used further for the subsequent experiments.

### *In vitro* Maturation and *in vitro* Fertilization

After warming, COCs were matured *in vitro* using in vitro maturation (IVM) medium for 17–18 hrs. IVM was performed in commercial medium (TCM-199; Invitrogen, Carlsbad, USA) supplemented with 20% FBS (Invitrogen), recombinant FSH/hCG (75 mIU/mL and 0.5 IU/mL) (Serono, Geneva, Switzerland), and recombinant epidermal growth factor (10 ng/mL; Sigma). After IVM, all COCs were denuded completely by treatment with 85 IU/mL hyaluronidase, and nuclear maturation was assessed. The extrusion of the first polar body was used as the maturation criterion and was scored under an inverted microscope (200×). After IVM, mature MII oocytes were fertilized with epididymal sperm. All protocols used were the same as those described previously [23].

**Table 3:** PCR primer sequences and product sizes.

Genes	Primer sequences(5′-3′)	Accessionnumber	Size (bp)	T_ann_ a(°C)
GAPDH	F^b^: accacagtccatgccatcac	BC092294	451	60
	R^c^: tccaccaccctgttgctgta			
Gdf9	F: ggttctatctgataggcgagg	NM008110	446	64
	R: ggggctgaaggagggagg			
Zp1	F: ccaatggccgtgtggat	NM009580	825	56
	R: ggtggttggggtgagaaga			
Zp2	F: agcccacattctgcccttga	NM011775	320	56
	R: gtcaattccattggcatgcc			
Zp3	F: aaatcggctcccaccttcca	NM011776	350	56
	R: ggctttgttgagcttatcgg			
Mater	F: gagcatcatggaggtgaagag	NM011860	300	56
	R: cttctggttaatcagcagcca			
Hsf1	F: acaacaacatggctagcttcg	AF082485	280	56
	R: ggagtccatacactcctgttt			
Zar1	F: caccaaagccggggatggctg	AY191415	330	55
	R: cggtctgccaggatcgcgggg			
Sod1	F: aaccatccacttcgagcagaa	NM011434	270	56
	R: gagtgagatcacacgatcttc			
Caspase3	F: aggggtcatttatgggaca	NM009810	422	59
	R: tacacgggatctgtttctttg			
Caspase6	F: ggcaaccacgtttacgcatac	NM009811	406	56
	R: ggcgctgagagacctttctgt			
Bcl2	F: taccgtcgtgacttcgcagag	NM009741	350	56
	R: ggcaggctgagcagggtctt			
Bax	F: cggcgaattggagatgaactg	NM007527	160	58
	R: gcaaagtagaagagggcaacc			
CIRP	F: ccgaggctttgggtttgt	NM007705	316	55
	R: atagctgccaccctgact			
Rbm3	F: aaagatcccggggttttggc	NM016809	300	55
	R: gtctctgtaatttcctcctg			
Mad2	F: tcagcgtggcatttatcc	NM019499	422	54
	R: attgcggtcccgattctt			
Eg5	F: caaccaccaatgatgctaaacag	NM010615	610	56
	R: gagcctccctctcttcatcca			
Hook1	F: tggaagaagagctgaagaagg	NM030014	320	56
	R: tgtattccacgggcatgatct			

a Annealing temperature. ^b^Forward primer. ^c^Reverse primer.

**Figure 4:**
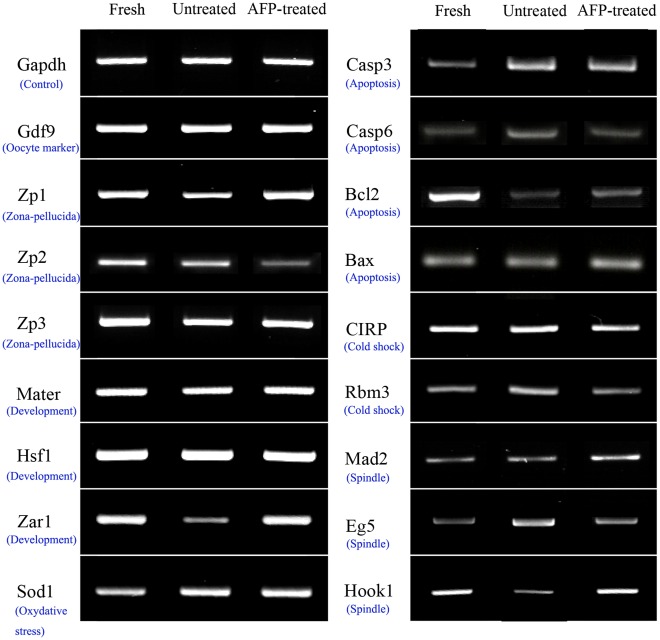
The expression of genes associated with meiotic or mitotic checkpoints (Mad2, Eg5), microtubule microstructure (Hook1), maternal effects (Mater, Hsf1, and Zar1), oxidative stress (Sod1), zona pellucida (Zp1, Zp2, and Zp3), apoptosis (Bcl2, Bax, Caspase3, and Caspase6) and cold-stress (cold-inducible RNA-binding protein [CIRP] and cold-shock protein RNA-binding motif protein-3 [Rbm3]) in vitrified-warmed, *in vitro* matured mouse oocytes (fresh, non-treated, and AFP-treated).

### Blastocyst Cell Counts and Caspase Staining

A part of the blastocyst was stained using the CaspaTag Pan-Caspase *in situ* assay kit according to the manufacturer’s instructions (Millipore, New Bedford, USA). Positive controls were incubated in 0.1% H_2_O_2_ for 1 min before staining. Negative controls were incubated in polyvinyl alcohol (PVA; Sigma)/PBS solution only. The blastocysts were mounted on slides and evaluated with fluorescence microscopy. Caspase-positive cells stained green, and all nuclei were stained blue. Caspase positivity was determined as the ratio of caspase-positive blastomeres to the total number of nuclei. (Figure 1, upper panel).

### Differential Staining of Blastocysts

The numbers of inner cell mass (ICM) and trophectoderm (TE) cells in the blastocysts were determined according to the method developed by Handyside and Hunter [44], with minor modifications. Briefly, the zona pellucida was removed from the blastocysts by culturing in mMTF containing 0.5% protease (Sigma) for 15 min at 37°C in humidified air containing 5% CO_2_. After rinsing with 0.4% PVA/PBS for 5 min, the naked blastocysts were transferred to HEPES-mMTF (1∶3) containing rabbit anti-mouse splenocyte antiserum (Sigma) and incubated in this solution for 20 min at 37°C in humidified air containing 5% CO_2_. After being washed 3 times with 0.4% PVA/PBS for 5 min each, the blastocysts were immersed in HEPES-mMTF (1∶5) containing guinea pig complement for another 20 min at 37°C in humidified air containing 5% CO_2_. After rinsing, the blastocysts were placed in a staining solution containing 5 µg/mL Hoechst 33342 and 5 µg/mL propidium iodide (PI, Sigma) in 0.4% PVA/PBS for 15 min at room temperature. The stained blastocysts were mounted on light microscope slides in droplets of mounting medium. The numbers of TE and ICM cells were counted under a fluorescence microscope (Leica DMIL; Leica Microsystems GmbH, Ernst-leitz-Strasse, Germany) with a Hamamatsu digital camera imaging system. The nuclei of the TE cells were stained red-pink, while the nuclei of the ICM cells were stained blue (Figure 1, lower panel).

### Meiotic Spindle and Chromosome Evaluation

After warming and in vitro maturation, matured oocytes were incubated for 2 hrs at 37°C in highly humidified air containing 5% CO2. Spindle integrity was assessed using previously described methods [45]. The localization of tubulin and chromatin revealed by FITC and DAPI fluorescence was observed under ×400 magnification with the use of a fluorescence microscope (Leica DMIL) with a Hamamatsu digital camera imaging system.

A typical barrel-shaped microtubule structure between both poles with centrally aligned chromosomes was considered normal. The meiotic spindles and chromosome alignments which were slightly damaged, for example some loss of spindle, were counted as sub-normal. Any other configurations were considered as abnormal (Figure 2).

### Membrane Integrity Evaluation

Warmed and *in vitro* matured oocytes were incubated in PBS supplemented with 5 mg/mL BSA, 1 mg/mL fluorescein diacetate (FDA, Sigma) and 50 mg/mL PI (Sigma) for 10 min [46]. Live cells accumulate intracellular fluorescein when exposed to FDA and thus appear green under fluorescence microscopy. PI is a potent nuclear stain that is generally excluded from live cells. Oocytes stained with FDA and PI simultaneously were considered to be alive but damaged (Figure 3).

### mRNA Isolation

mRNA was extracted from five *in vitro* matured oocytes from each experimental sample using the Dynabeads mRNA DIRECT micro kit (Dynal Asa, Oslo, Norway) according to the manufacturer’s instructions.

### RT-PCR Analysis

cDNA was synthesized from mRNA using 50 ng/mL random hexamer primers, according to the SuperScript Preamplification System protocol (Gibco-BRL, Grand Island, USA). PCR was carried out according to the Nova Taq amplification protocol (Nova Clean-Taq, Genenmed, Korea). Five oocyte-equivalents of cDNA was used as the template for PCR analysis. PCR conditions and primer sequences are listed in Table 3. The expression levels of meiotic and mitotic checkpoint-related genes (Mad2 and Eg5), a microtubule structure gene (Hook1), a radical oxygen species-related gene (Sod1), maternally derived genes (Mater, Hsf1, and Zar1), zona pellucida genes (Zp1, Zp2, and Zp3), Bcl2/Bax, Caspase3/Caspase6, cold-inducible RNA-binding protein (CIRP), and cold-shock protein RNA-binding motif protein-3 (Rbm3) were analyzed in cDNA prepared from three groups of oocytes: fresh control, AFP-treated, and untreated (Figure 4). All experiments were repeated three times. GAPDH and oocyte-specific Gdf9 were used as internal controls.

### Statistical Analyses

Data were analyzed with SPSS (v.17, Chicago, IL, USA). Ratios were compared using Chi-squared tests. The means were compared using unpaired Student’s t-tests. P-values <0.05 were considered statistically significant.

## References

[1] Ubaldi F, Anniballo R, Romano S, Baroni E, Albricci L (2010). Cumulative ongoing pregnancy rate achieved with oocyte vitrification and cleavage stage transfer without embryo selection in a standard infertility program.. Hum Reprod.

[2] Cobo A, Meseguer M, Remohi J, Pellicer A (2010). Use of cryo-banked oocytes in an ovum donation programme: a prospective, randomized, controlled, clinical trial.. Hum Reprod.

[3] Cobo A, Romero JL, Perez S, de los Santos MJ, Meseguer M (2010). Storage of human oocytes in the vapor phase of nitrogen.. Fertil Steril.

[4] Nagy ZP, Chang CC, Shapiro DB, Bernal DP, Kort HI (2009). The efficacy and safety of human oocyte vitrification.. Semin Reprod Med.

[5] Smith GD, Serafini PC, Fioravanti J, Yadid I, Coslovsky M (2010). Prospective randomized comparison of human oocyte cryopreservation with slow-rate freezing or vitrification.. Fertil Steril.

[6] Rao GD, Chian RC, Son WS, Gilbert L, Tan SL (2004). Fertility preservation in women undergoing cancer treatment.. Lancet.

[7] Isachenko E, Rahimi G, Isachenko V, Nawroth F (2004). In-vitro maturation of germinal-vesicle oocytes and cryopreservation in metaphase I/II: a possible additional option to preserve fertility during ovarian tissue cryopreservation.. Reprod Biomed Online.

[8] Cooper A, Paynter SJ, Fuller BJ, Shaw RW (1998). Differential effects of cryopreservation on nuclear or cytoplasmic maturation in vitro in immature mouse oocytes from stimulated ovaries.. Hum Reprod.

[9] Isachenko EF, Nayudu PL (1999). Vitrification of mouse germinal vesicle oocytes: effect of treatment temperature and egg yolk on chromatin and spindle normality and cumulus integrity.. Hum Reprod.

[10] Toth TL, Lanzendorf SE, Sandow BA, Veeck LL, Hassen WA (1994). Cryopreservation of human prophase I oocytes collected from unstimulated follicles.. Fertil Steril.

[11] Toth TL, Baka SG, Veeck LL, Jones HW, Muasher S (1994). Fertilization and in vitro development of cryopreserved human prophase I oocytes.. Fertil Steril.

[12] Son WY, Park SE, Lee KA, Lee WS, Ko JJ (1996). Effects of 1,2-propanediol and freezing-thawing on the in vitro developmental capacity of human immature oocytes.. Fertil Steril.

[13] Yeh Y, Feeney RE (1996). Antifreeze Proteins: Structures and Mechanisms of Function.. Chem Rev.

[14] Fletcher GL, Hew CL, Davies PL (2001). Antifreeze proteins of teleost fishes.. Annu Rev Physiol.

[15] Arav A, Rubinsky B, Fletcher G, Seren E (1993). Cryogenic protection of oocytes with antifreeze proteins.. Mol Reprod Dev.

[16] Payne SR, Oliver JE, Upreti GC (1994). Effect of antifreeze proteins on the motility of ram spermatozoa.. Cryobiology.

[17] Madura JD, Baran K, Wierzbicki A (2000). Molecular recognition and binding of thermal hysteresis proteins to ice.. J Mol Recognit.

[18] Rubinsky B, Arav A, Mattioli M, Devries AL (1990). The effect of antifreeze glycopeptides on membrane potential changes at hypothermic temperatures.. Biochem Biophys Res Commun.

[19] Habibi A, Farrokhi N, Moreira da Silva F, Bettencourt BF, Bruges-Armas J (2010). The effects of vitrification on gene expression in mature mouse oocytes by nested quantitative PCR.. J Assist Reprod Genet.

[20] Musacchio A, Hardwick KG (2002). The spindle checkpoint: structural insights into dynamic signalling.. Nat Rev Mol Cell Biol.

[21] Wang WH, Sun QY (2006). Meiotic spindle, spindle checkpoint and embryonic aneuploidy.. Front Biosci.

[22] Wang JZ, Sui HS, Miao DQ, Liu N, Zhou P (2009). Effects of heat stress during in vitro maturation on cytoplasmic versus nuclear components of mouse oocytes.. Reproduction.

[23] Jo JW, Jee BC, Lee JR, Suh CS (2011). Effect of antifreeze protein supplementation in vitrification medium on mouse oocyte developmental competence..

[24] Hagan I, Yanagida M (1990). Novel potential mitotic motor protein encoded by the fission yeast cut7+ gene.. Nature.

[25] Hoyt MA, He L, Loo KK, Saunders WS (1992). Two Saccharomyces cerevisiae kinesin-related gene products required for mitotic spindle assembly.. J Cell Biol.

[26] Blangy A, Lane HA, d’Herin P, Harper M, Kress M (1995). Phosphorylation by p34cdc2 regulates spindle association of human Eg5, a kinesin-related motor essential for bipolar spindle formation in vivo.. Cell.

[27] Castillo A, Justice MJ (2007). The kinesin related motor protein, Eg5, is essential for maintenance of pre-implantation embryogenesis.. Biochem Biophys Res Commun.

[28] Castillo A, Morse HC, Godfrey VL, Naeem R, Justice MJ (2007). Overexpression of Eg5 causes genomic instability and tumor formation in mice.. Cancer Res.

[29] Simpson F, Martin S, Evans TM, Kerr M, James DE (2005). A novel hook-related protein family and the characterization of hook-related protein 1.. Traffic.

[30] Hamatani T, Falco G, Carter MG, Akutsu H, Stagg CA (2004). Age-associated alteration of gene expression patterns in mouse oocytes.. Hum Mol Genet.

[31] Tong ZB, Gold L, Pfeifer KE, Dorward H, Lee E (2000). Mater, a maternal effect gene required for early embryonic development in mice.. Nat Genet.

[32] Christians E, Davis AA, Thomas SD, Benjamin IJ (2000). Maternal effect of Hsf1 on reproductive success.. Nature.

[33] Wu X, Viveiros MM, Eppig JJ, Bai Y, Fitzpatrick SL (2003). Zygote arrest 1 (Zar1) is a novel maternal-effect gene critical for the oocyte-to-embryo transition.. Nat Genet.

[34] Blomberg LA, Long EL, Sonstegard TS, Van Tassell CP, Dobrinsky JR (2005). Serial analysis of gene expression during elongation of the peri-implantation porcine trophectoderm (conceptus).. Physiol Genomics.

[35] Wassarman PM (1988). Zona pellucida glycoproteins.. Annu Rev Biochem.

[36] Wassarman PM (1988). Fertilization in mammals.. Sci Am.

[37] Hinsch E, Groeger S, Oehninger S, Hinsch KD (2003). Localization and functional importance of a conserved zona pellucida 2 protein domain in the human and bovine ovary using monoclonal anti-ZP2 peptide antibodies.. Theriogenology.

[38] Wrenzycki C, Herrmann D, Carnwath JW, Niemann H (1999). Alterations in the relative abundance of gene transcripts in preimplantation bovine embryos cultured in medium supplemented with either serum or PVA.. Mol Reprod Dev.

[39] Yang MY, Rajamahendran R (2002). Expression of Bcl-2 and Bax proteins in relation to quality of bovine oocytes and embryos produced in vitro.. Anim Reprod Sci.

[40] Izawa M, Nguyen PH, Kim HH, Yeh J (1998). Expression of the apoptosis-related genes, caspase-1, caspase-3, DNA fragmentation factor, and apoptotic protease activating factor-1, in human granulosa cells.. Fertil Steril.

[41] Exley GE, Tang C, McElhinny AS, Warner CM (1999). Expression of caspase and BCL-2 apoptotic family members in mouse preimplantation embryos.. Biol Reprod.

[42] Nishiyama H, Itoh K, Kaneko Y, Kishishita M, Yoshida O (1997). A glycine-rich RNA-binding protein mediating cold-inducible suppression of mammalian cell growth.. J Cell Biol.

[43] Zhou KW, Zheng XM, Yang ZW, Zhang L, Chen HD (2009). Overexpression of CIRP may reduce testicular damage induced by cryptorchidism.. Clin Invest Med.

[44] Handyside AH, Hunter S (1984). A rapid procedure for visualising the inner cell mass and trophectoderm nuclei of mouse blastocysts in situ using polynucleotide-specific fluorochromes.. J Exp Zool.

[45] Huang JY, Chen HY, Tan SL, Chian RC (2007). Effect of choline-supplemented sodium-depleted slow freezing versus vitrification on mouse oocyte meiotic spindles and chromosome abnormalities.. Fertil Steril.

[46] Somfai T, Dinnyes A, Sage D, Marosan M, Carnwath JW (2006). Development to the blastocyst stage of parthenogenetically activated in vitro matured porcine oocytes after solid surface vitrification (SSV).. Theriogenology.

