# Correction: The Beneficial Effects of Antifreeze Proteins in the Vitrification of Immature Mouse Oocytes

**DOI:** 10.1371/annotation/efb34647-9ddd-4860-b763-07e7c34dc89d

**Published:** 2014-01-17

**Authors:** Jun Woo Jo, Byung Chul Jee, Chang Suk Suh, Seok Hyun Kim

Figure 2 is incorrect. The correct version of Figure 2 can be viewed here: http://plosone.org/corrections/pone.0037043.g002.cn.tif

